# Subcutaneous Emphysema and Pneumomediastinum after Tonsillectomy

**DOI:** 10.1155/2013/154857

**Published:** 2013-11-26

**Authors:** George Koukoutsis, Dimitrios G. Balatsouras, Panayotis Ganelis, Alexandros Fassolis, Antonis Moukos, Michael Katotomichelakis, Antonis Kaberos

**Affiliations:** ^1^ENT Department, Tzanion General Hospital, Afentouli 1 & Zanni, 18536 Piraeus, Greece; ^2^ENT Department, Medical School, Democritus University of Thrace, University Hospital of Alexandroupolis, Dragana, 68100 Alexandroupoli, Greece

## Abstract

Cervicofacial subcutaneous emphysema is a rare complication of tonsillectomy that often resolves spontaneously but may progress to obstruct upper airways or spread to the thorax causing pneumomediastinum or pneumothorax. The mechanisms by which subcutaneous emphysema and pneumomediastinum may develop after tonsillectomy are poorly understood. A case of a 21-year-old female undergoing routine adenotonsillectomy, who developed cervicofacial emphysema and pneumomediastinum, is presented. Possible pathogenetic mechanisms and treatment options are discussed.

## 1. Introduction

Tonsillectomy is a common procedure performed in all otolaryngology departments. Hemorrhage remains the most significant risk, and other common complications include otalgia, odynophagia, damage to teeth, throat infection, nausea, and vomiting [1]. However, several rare complications have been reported, such as intraoperative vascular injury, Eagle syndrome, mediastinitis, atlantoaxial subluxation, cervical osteomyelitis, and taste disorders [2]. One of the most rare complications is subcutaneous emphysema of the neck. The aim of this report is to present a patient who presented with subcutaneous emphysema and pneumomediastinum after being submitted to palatine tonsillectomy and to discuss the possible pathogenetic mechanisms, morbidity, methods of prevention, and treatment of this potentially dangerous condition.

## 2. Case Report

A 21-year-old female patient was submitted to programmed palatine tonsillectomy with diagnosis of relapsing peritonsillar abscess. Preoperative workup including chest radiography and electrocardiography was normal. She went on surgery under general anesthesia with orotracheal intubation. Standard surgical technique was used, and dissection of palatine tonsil was performed on the subcapsular plane to avoid excessive trauma. Hemostasis was performed by means of bipolar electrocoagulation. The surgical procedure progressed uneventfully, with minimal bleeding, but the dissection was difficult because of the adhesion of the tonsils to the tonsillar bed. The patient recovered from anesthesia well, using oxygen mask for a while.

During the first postoperative hours, the patient presented cough and had an episode of emesis. Later, in the afternoon of the same day, the patient complained of pain in the right submandibular area. A physical examination revealed right mild, painless swelling of the right submandibular area. Inspection of the oral cavity did not evidence any bleeding from the tonsillar beds or the presence of any mucosal tear. No signs of airway or ventilator distress were evident. The patient was administered analgesics and food intake was forbidden. However, during a few hours further swelling was noticed, extending upwards to the right facial area and downwards to the lateral right cervical area. On palpitation the swelling was soft and painless, but characteristic crepitus was evident. 

**Figure 1:**
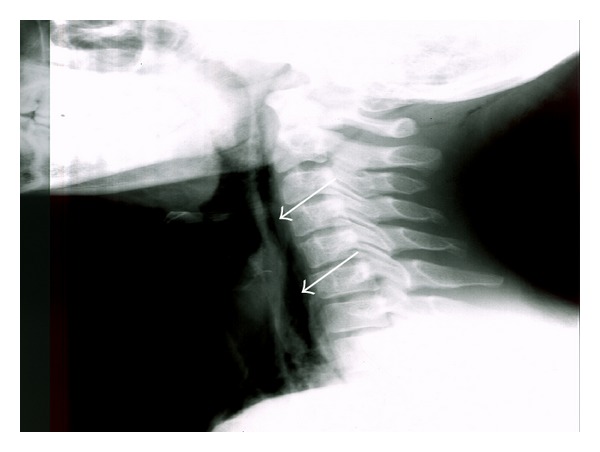
Lateral radiograph of the neck showing extensive subcutaneous emphysema (arrows).

**Figure 2:**
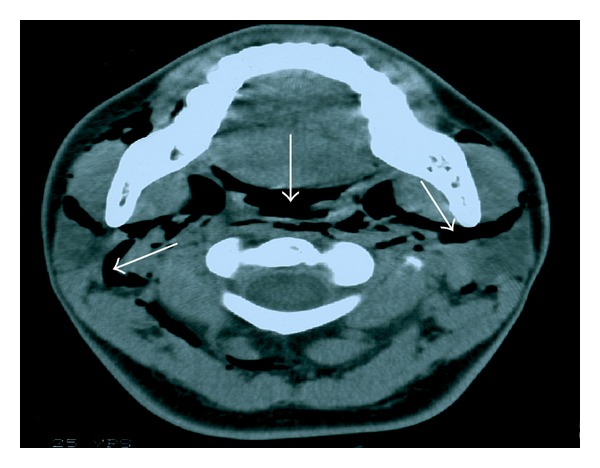
Axial computed tomography of the neck at the level of the inferior alveolar ridge reveals subcutaneous emphysema. The arrows indicate the presence of air in the neck.

**Figure 3:**
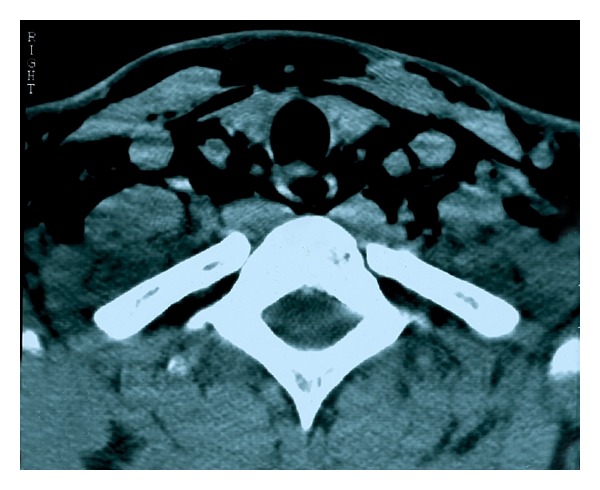
Axial computed tomography of the upper chest at the level of thyroid reveals the presence of pneumomediastinum.

**Figure 4:**
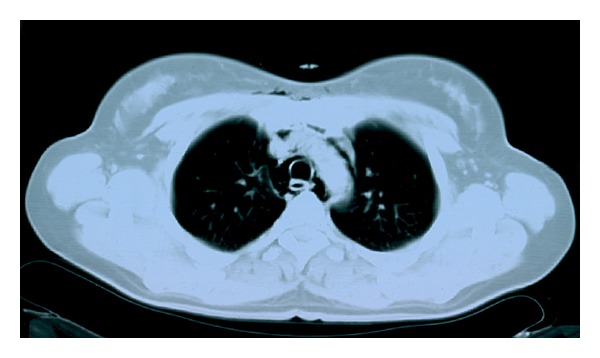
Axial computed tomography of the chest at the level of the aortic arch reveals the presence of air in the mediastinum.

A neck and chest X-ray revealed subcutaneous cervical emphysema (Figure 1) and a computed tomography scan of the neck (Figure 2) confirmed the diagnosis. A chest computed tomography performed simultaneously showed the presence of pneumomediastinum (Figures 3 and 4). Since the patient did not present with respiratory distress and hemodynamically was stable, conservative management was decided. A combination of broad-spectrum antibiotics was administered and the patient was restricted to bed rest and instructed to refrain from coughing. Further course was uneventful, the subcutaneous emphysema decreased substantially, and a new chest plain radiography showed absence of air. The patient was discharged a week after surgery and follow-up examinations did not reveal any abnormal findings. 

## 3. Discussion

Subcutaneous emphysema can occur either as a result of pressure difference across disruption in a mucosal surface or by the release of gas by organisms into an enclosed space [3]. Subcutaneous emphysema of the neck is usually caused by the disruption of the tracheobronchial tree or the esophagus. However, it can also occur, occasionally, after a break of the oropharyngeal mucosa, which can be secondary to surgical or anesthetic trauma [4]. 

Subcutaneous emphysema, with or without pneumomediastinum, is an extremely unusual complication of adenotonsillar surgery [5–8]. The mechanism by which free air may gain entry into the soft tissue planes of the neck is a matter of speculation. Most authors agree that the most likely portal of entrance of air into the soft tissues of the neck is from a tear of the tonsillar bed, secondary to the surgical procedure [8, 9]. Postoperative factors that may facilitate this include retching, vomiting, and coughing by which air may be forced against a blocked oronasopharynx, through the mucosal tear, into the cervical soft tissues. In a case described by Podoshin et al. [10], histologic examination detected some striped-muscle bundles attached to the tonsil, which indicated damage to the tonsillar bed. In our patient, although coughing and vomiting occurred during the first postoperative hours, no presence of any mucosal break was evident either on inspection or in the computed tomography scan performed later. However, since we had difficulty dissecting the tonsil from the tonsillar bed because of severe adhesions, the presence of lesser tears of the mucosa should not be ruled out, explaining the entrance of air into the neck tissues, especially after coughing and vomiting. 

Other possible pathogenetic mechanisms include a rupture anywhere along a defect of the tracheobronchial tree [11], resulting in a primary pneumomediastinum and decompression in a cephalad direction, extending in secondary involvement of the soft tissues of the neck. This defect in the tracheobronchial tree may be a preexisting abnormality, such as an alveolar bleb or laryngocele or may be an acquired defect in the laryngotracheal mucosa from a traumatic intubation [12]. When such a defect is present, rupture may occur secondary to excessive positive pressure ventilation or excessive manual ventilation. It may also be attributed to a sharp increase in intrathoracic pressure against a total or partial obstruction, such as coughing, straining, or vomiting, usually as the patient awakens from general anesthesia. 

The severity of this complication can range from a self-resolving emphysema limited to the neck to an extension over the chest and significant respiratory problems [7, 11]. The subcutaneous emphysema may also predispose for mediastinitis and necrotizing fasciitis of the neck. Its main clinical characteristic is crepitus, and the subcutaneous air can be readily detected by a chest X-ray [3, 8]. Treatment of subcutaneous emphysema varies according to level of severity [7]. Treatment should be conservative in the majority of cases and based on the nature and benign course of the disease. A few days of regular assessment of the extent of the emphysema and of the airway are needed. Any activity that increases upper airway pressure such as coughing, vomiting, and straining should be avoided. Broad-spectrum antibiotics may also be prescribed. Additionally, to prevent the secondary entrance of bacteria to the subcutaneous emphysema and to limit the extension of surgical emphysema, the injured mucosa may be sutured, when a disruption is evident. In rare cases, a tracheotomy or even a thoracotomy has been required [7, 13]. 

It may be thus concluded that although subcutaneous emphysema resulting as a complication of tonsillectomy is rare and usually benign, early diagnosis and appropriate supportive measures are necessary to prevent its further extension, which could be accompanied by respiratory distress.
